# Thymidine Phosphorylase Is Increased in COVID-19 Patients in an Acuity-Dependent Manner

**DOI:** 10.3389/fmed.2021.653773

**Published:** 2021-03-22

**Authors:** Wei Li, Hong Yue

**Affiliations:** Department of Biomedical Sciences, Joan C. Edwards School of Medicine of Marshall University, Huntington, WV, United States

**Keywords:** COVID-19, SARS-CoV-2, thymidine phosphorylase, thrombosis, acuity, tipiracil

## Abstract

Coronavirus disease 2019 (COVID-19), caused by the severe acute respiratory syndrome coronavirus 2 (SARS-COV-2), is a human respiratory disease. Hitherto, there is no effective treatment has been established. Patients with cardiovascular or diabetes comorbidities are a high-risk cohort. COVID-19 is accompanied by excessive systemic thrombotic events, but the mechanism is not yet known. Recent studies have indicated that thymidine phosphorylase (TYMP) plays an important role in platelet activation, thrombosis, and TYMP expression is increased in diabetic patients. By using data provided by the MGH (Massachusetts General Hospital) Emergency Department COVID-19 Cohort with Olink Proteomics, here we show that plasma TYMP level is correlated with the COVID-19 associated thrombotic event, inflammation, and organ damage, as evidenced by the positive correlations with plasma D-dimer, CRP (C reactive protein), and LDH (lactate dehydrogenase), as well as Interferons (IFN). Plasma TYMP is also positively correlated with COVID-19 patients who had respiratory symptoms. TYMP thus could be an acuity marker for COVID-19 diagnosis. Targeting TYMP with tipiracil, a selective TYMP inhibitor, which has been approved by the Food and Drug Administration for clinical use, could be a novel effective medicine for COVID-19.

## Background

COVID-19 is a respiratory illness caused by severe acute respiratory syndrome coronavirus 2 (SARS-CoV-2) (1). Large-scale reports have characterized the symptoms, comorbidities, and clinical outcomes, and patients with cardiovascular- or diabetes-associated comorbidities are at a higher risk to develop advanced COVID-19 and need admission to intensive care (ICU) (2–5). While COVID-19 vaccines are distributing around the world, the long-term efficiency is unknown. We still do not know when we are able to achieve herd immunity and control morbidity and mortality at an acceptable rate. Currently, no effective regimen for the COVID-19 has been established, and an interim WHO (World Health Organization) solidarity trial report indicated that remdesivir, hydroxychloroquine, lopinavir, and interferon regimens have little or no effect on the hospitalized COVID-19 patients (6). Patients with advanced COVID-19 often die from acute respiratory distress syndrome or multiorgan failure (7). Accumulating evidence implicates that inflammatory cytokine storm plays an important role in the COVID-19 milieu (8). About 71% of the non-surviving COVID-19 patients had disseminated intravascular coagulation (9). SARS-CoV-2 positive individuals have an increased risk of heart attack and stroke (10). These data suggest that some types of thrombotic events happened concurrently with the COVID-19. However, the cause of COVID-19-associated thrombotic events is uncertain.

TYMP catalyzes the reversible conversion of thymidine to thymine and 2-deoxy-D-ribose-1 phosphate and plays an important role in the pyrimidine salvage pathway (11). TYMP is also known as platelet-derived endothelial cell growth factor and is highly expressed in human platelets (11, 12). Our recent study, for the first time, demonstrated that TYMP has a signaling function in platelets and plays a mechanistic role in platelet activation and thrombosis (12, 13). Inhibition of TYMP with tipiracil hydrochloride (TPI), a selective, potent, and Food and Drug Administration-approved TYMP inhibitor, dramatically inhibits thrombosis in mice without increasing the risk of bleeding (13). Several inflammatory cytokines, including TNFα, IL-1, IL-6, IL-8, and IFN-γ induce TYMP expression (11, 14–16). TYMP also enhances the expression of IL-8 and CXCL10 (15, 17). All of these cytokines form the component of inflammatory cytokine storm associated with COVID-19 (8, 18, 19). TYMP expression was increased in diabetic patients (20, 21), which are a high-risk cohort to develop severe COVID-19. Based on these pre-clinical and clinical studies, we tested the hypothesis that TYMP plays an important role in the SARS-CoV-2-induced inflammation and the COVID-19 associated thrombosis.

## Methods

We used the database provided by the MGH (Massachusetts General Hospital) emergency department COVID-19 Cohort (Filbin, Goldberg, Hacohen) with Olink Proteomics (22), which includes four 384-plex panels focused on inflammation, oncology, cardiometabolic, and neurology proteins. TYMP was only found in the cardiometabolic panel. Totally, 733 TYMP data points were extracted. The TYMP data were stratified based on the final diagnoses: COVID-19 negative or positive. Both groups were further stratified based on age, BMI (body mass index), days after hospitalization, acuity assessed based on WHO Ordinal Outcomes Score, levels of plasma D-dimer, absolute counts of nucleated cells, plasma CRP (C-reactive protein), and LDH (lactate dehydrogenase) levels, as well as fever. The data were also stratified based on the presence or absence of pre-existing disease and respiratory symptoms, including sore throat, congestion, productive or dry cough, shortness of breath or hypoxia, or chest pain. See Supplementary Materials for detailed information. TYMP expression was then analyzed and compared among the defined groups. The data were analyzed using the 2-tailed Student's *t-*test with GraphPad Prism (version 9.0.0) and expressed as mean ± SD. The Pearson correlation coefficient was used for analyzing the correlation between TYMP and IFNs. A *p* ≤ 0.0*5* was considered statistically significant.

## Results

**Figure 1:**
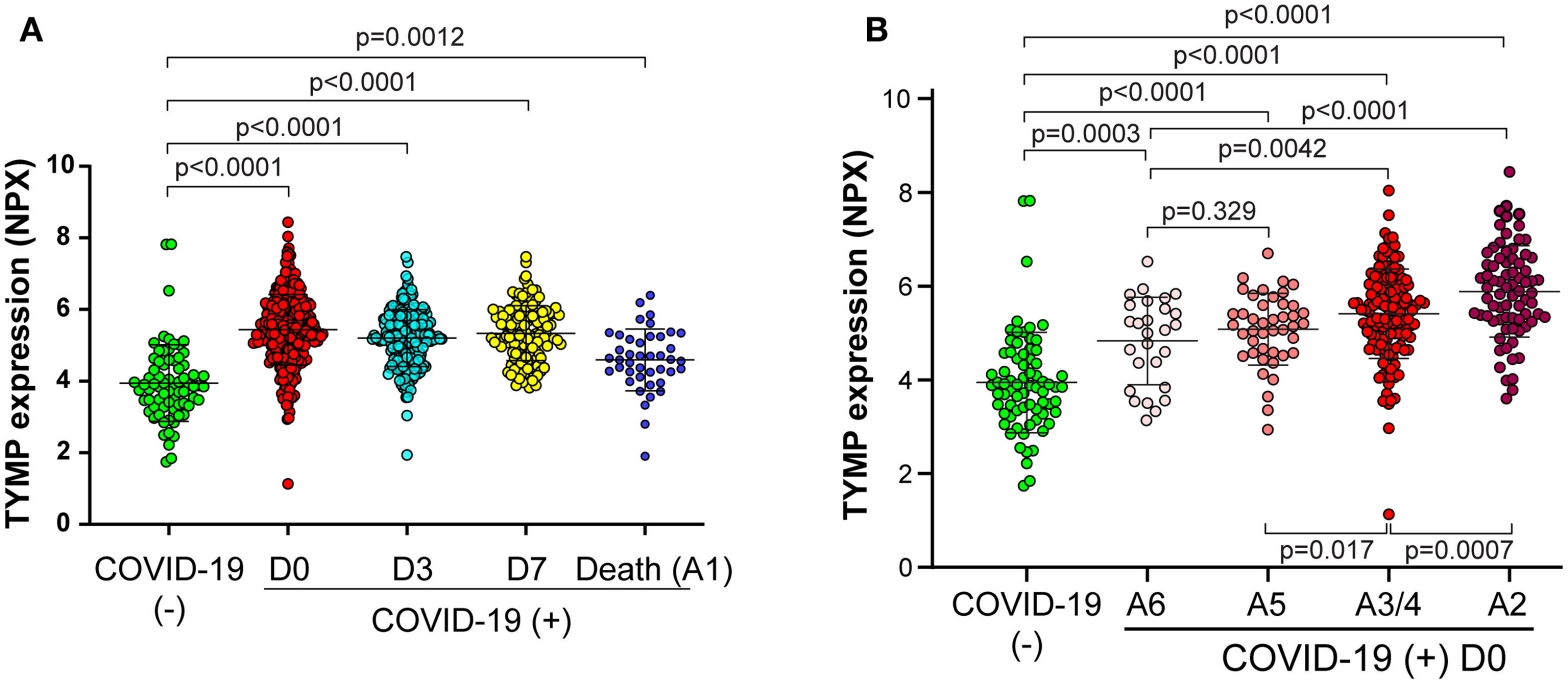
Plasma TYMP level is correlated with the acuity of patient with COVID-19. TYMP data were extracted from the original database provided by the MGH (Massachusetts General Hospital) Emergency Department COVID-19 Cohort with Olink Proteomics. Data were sorted based on days after hospitalization **(A)** and acuity **(B)**, and then TYMP expression was analyzed among the defined groups. Day (D) 0, 3, and 7 = enrollment plus 1 day, 3 days, and 7 days. Acuity (A) 1 = Death within 28 days, A2 = Intubated, ventilated, survived to 28 days, A3 = Non-invasive ventilation or high-flow nasal cannula, A4 = Hospitalized, supplementary O_2_ required, A5 = Hospitalized, no supplementary O_2_ required, and A6 = Not hospitalized.

In the MGH study, totally, 384 patients with acute respiratory distress, which was defined with at least one of the following symptoms: (1) tachypnea (≥22 breaths per minute), (2) oxygen saturation ≤92% on room air, (3) a requirement for supplemental oxygen, or (4) positive pressure ventilation, were enrolled in the Emergency Department from 3/24/2020 to 4/30/2020 (22). Among them, 78 patients were confirmed SARS-CoV-2 negative and 306 were positive. These 78 non-COVID-19 patients were categorized as controls. Samples collected from 358 patients (284 are COVID-19) on day 0, 202 patients (197 are COVID-19) on day 3, and 131 patients (all are COVID-19) on day 7, as well as 42 patients who died from COVID-19 within 28 days, were extracted for analyzing TYMP expression. We found plasma TYMP were significantly increased in COVID-19 patients on day 0, 3, and 7 when compared with non-COVID-19 patients (Figure 1A). Patients who died from COVID-19 also had a higher level of plasma TYMP compared with non-COVID-19 patients. Since we do not know when these patients died and when their plasma samples were harvested, we excluded this group from the following analysis.

By stratifying patients by acuity levels on day 0, 3, and 7, based on the WHO Ordinal Outcomes Score, we found TYMP expression showed an acuity-dependent manner on day 0 (Figure 1B). In comparing with non-COVID-19 patients, COVID-19 patients, even those who were discharged from the emergency department (A6) had a significantly high level of plasma TYMP (*p* = 0.0003, Figure 1B). Among the hospitalized patients, patients who did not need supplemental oxygen (A5) had the lowest, patients who needed oxygen support (A3/4) had the middle level, and patients who received intubation (A2) had the highest level of plasma TYMP. On day 3, patients who received intubation still have a higher plasma TYMP when compared with patients without the need of supplementary oxygen (Supplementary Figure 1A). The acuity-associated TYMP expression was the same on day 7 (Supplementary Figure 1B). These data suggest that plasma TYMP are positively correlated with the acuity of COVID-19, especially in the early phase. We thus further analyzed the correlation between TYMP and COVID-19 associated severity markers on day 0.

**Figure 2:**
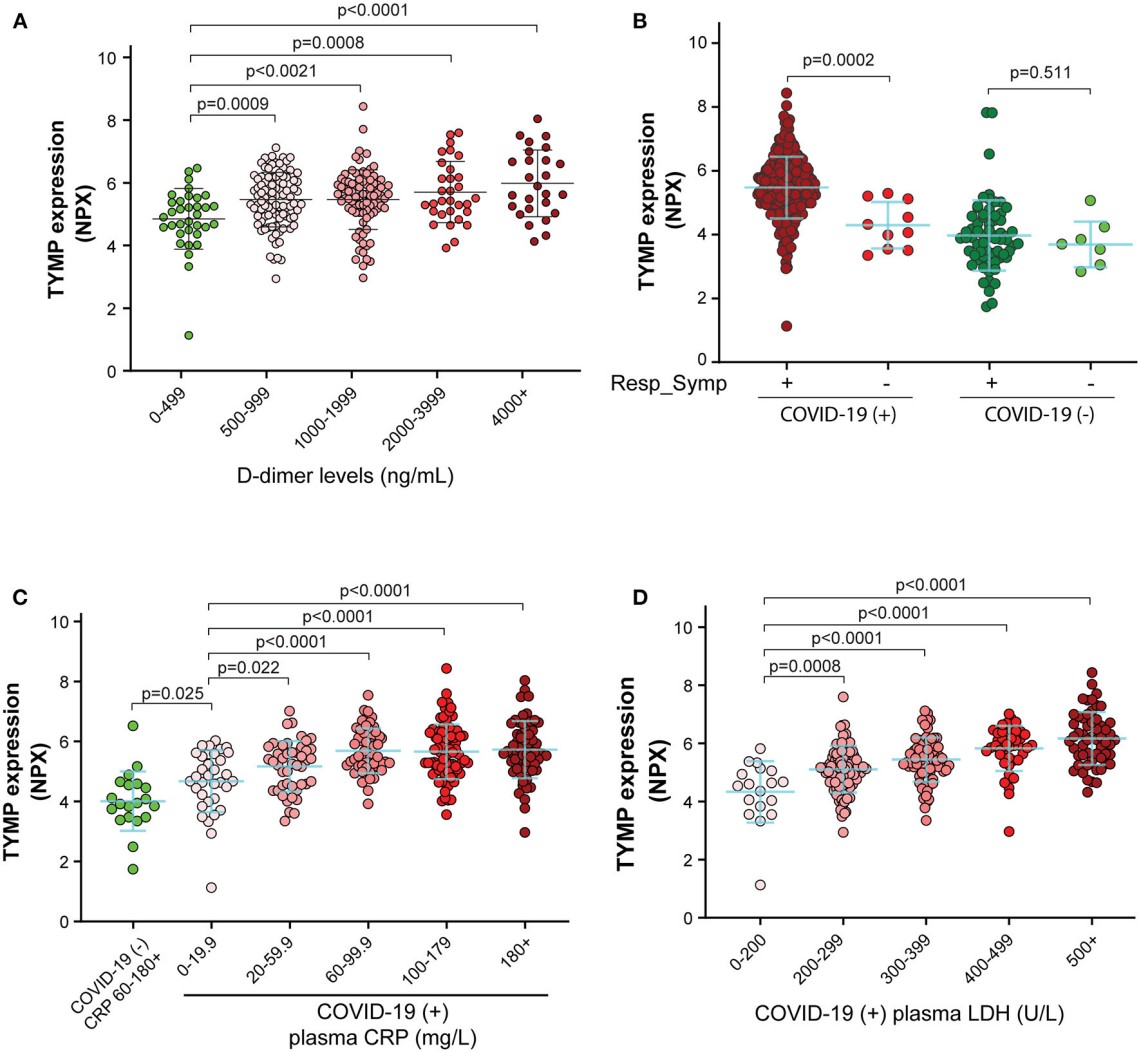
Plasma TYMP level is correlated with the thrombotic event, the presence of respiratory symptoms, inflammation, and tissue damage in patients with COVID-19. TYMP data were sorted based on plasma levels of D-dimer **(A)**, the presence or absence of respiratory symptoms **(B)**, CRP (C-reactive protein) **(C)**, and LDH (lactate dehydrogenase) **(D)**, and then TYMP expression was analyzed based on the defined groups.

COVID-19 patients have reduced platelet counts and elevated plasma D-dimer (9), suggesting that an excessive thrombotic event occurred. Since TYMP plays a mechanistic role in platelet activation and thrombosis (11–13), we analyzed the correlation between plasma TYMP and D-dimer. We found that the higher the D-dimer concentration the patients had, the more plasma TYMP were detected (Figure 2A). These data suggest that TYMP is correlated with the COVID-19 associated thrombotic event. TYMP is also expressed in nucleated cells (23), we thus further analyzed TYMP expression based on the absolute counts of monocyte, lymphocyte, and neutrophil. As shown in Supplementary Figures 2A–C, TYMP expression was not associated with the count of these nucleated cells. TYMP expression was also not associated with fever in COVDI-19 either positive or negative patients (Supplementary Figure 2D).

Among the MGH COVID-19 Cohort, TYMP expression was not associated with age and BMI in both COVID-19 positive and negative patients (Supplementary Figures 3A–D). Primary kidney diseases, hypertension, and pre-existing immunocompromised conditions, as well as pre-existing gastrointestinal diseases and diabetes also did not affect TYMP expression in both COVID-19 positive and negative patients (Supplementary Figures 3E–I). These data suggest that TYMP expression is not affected by age or the presence of some diseased conditions. In non-COVID-19 patients, TYMP expression was similar between patients with and without heart or lung disease. However, TYMP expression at day 0 was lower in the COVID-19 patients who had primary heart or lung diseases (Supplementary Figures 4A,B). The acuity assessed using the WHO Ordinal Outcomes Score for day 0 was not different between patients with and without heart or lung disease, suggesting that the primary lung or heart disease associated low-TYMP expression in the patients with COVID-19 does not reflect the acuity. TYMP expression was similar in SARS-CoV-2 negative patients when they were divided into two groups based on whether they have respiratory symptoms or not (Figure 2B). However, TYMP expression was significantly increased in COVID-19 patients with respiratory symptoms when compared with COVID-19 patients without respiratory symptoms (Figure 2B). These data suggest that in addition to the thrombotic event, lung injury is also associated with plasma TYMP levels.

CRP is a critical component of the immune system and is a predictive factor for inflammation and future risk of cardiovascular events. We found patients who had a higher CRP level also had a higher plasma TYMP (Figure 2C). In the non-COVID-19 patients, even their plasma level of CRP was above 60–180 mg/L, their TYMP expression was still significantly lower than in the COVID-19 patients with CRP < 20 mg/L (Figure 2C, *p* = 0.025). Similar to CRP, TYMP expression was also significantly correlated with the plasma level of LDH, an indicator of tissue damage (Figure 2D).

**Figure 3:**
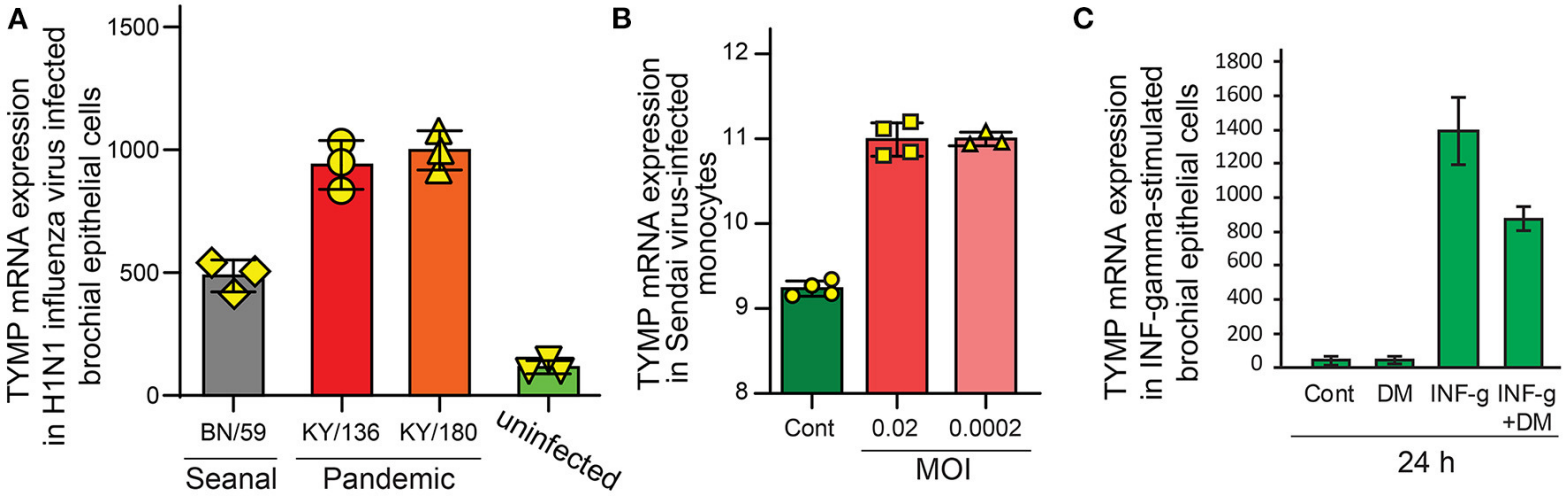
TYMP expression is upregulated by virus infection and is associated with inflammation. GEO profile data were searched using keywords “TYMP + Virus.” **(A)** (GDS4855). Human bronchial epithelial cells were infected with seasonal influenza virus (BN/59), H1N1 pandemic virus isolated from two individuals (KY/136 and KY/180), and uninfected cells were used as control. **(B)** (GSE67198). U937 cells were uninfected or infected with either 0.02 or 0.0002 multiplicity of infection (MOI) Sendai virus for 24 h. **(C)** (GDS1256). Human bronchial epithelial cells were treated with IFN-γ, dexamethasone, or their combination for 24 h.

**Figure 4:**
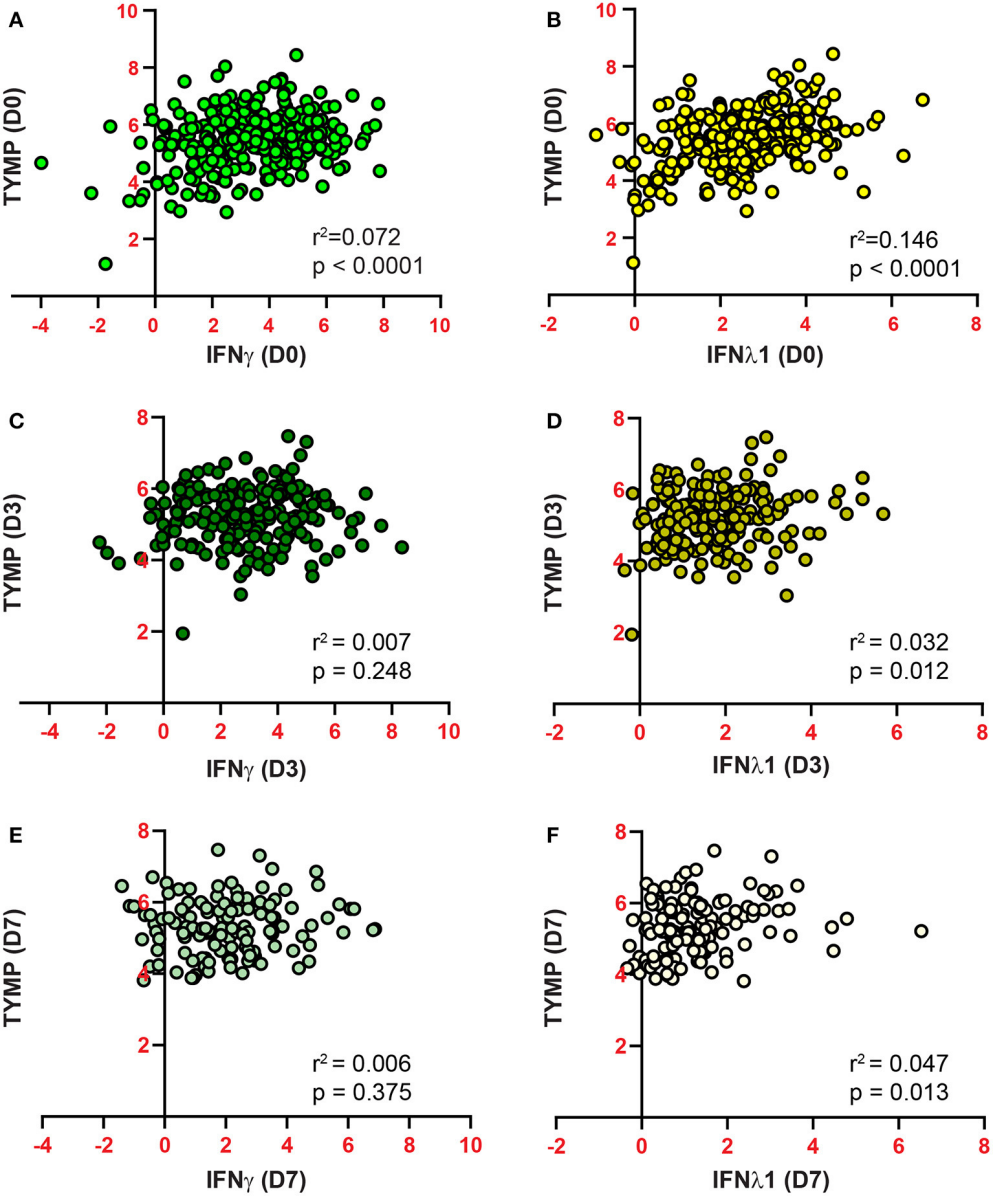
Plasma TYMP levels are correlated with the expression of IFN-γ **(A,C,D)** and IFN-λ **(B,D,F)** in COVID-19 patients. TYMP, IFN-γ, and IFN-λ data on day 0 **(A,B)**, 3 **(C,D)**, and 7 **(E,F)** were extracted and the correlation between plasma TYMP levels and IFN-γ or IFN-λ was analyzed, respectively.

By searching GEO (Gene Expression Omnibus) profile using keywords “TYMP + virus,” we found that TYMP was also upregulated in several other viral infections, including influenza virus H1N1 infected bronchia epithelial cells (Figure 3A) (24) and Sendai-virus infected monocytes (Figure 3B) (25). IFN-γ, which was upregulated by SARS-CoV-2 infection (8, 18), significantly increased TYMP mRNA expression in the primary cultured bronchial epithelial cells (Figure 3C). The IFN-γ-induced TYMP expression was inhibited by dexamethasone, an anti-inflammatory steroid (26). We thus further analyzed the correlation between plasma IFN and TYMP levels. INF was found in the inflammation panel, and only IFN-γ and IFN-λ were detected. As shown in Supplementary Figure 5, there was no correlation between plasma TYMP with either IFN-γ or IFN-λ in the non-COVID-19 patients on day 0. However, in the COVID-19 patients, a positive correlation was found between IFN-γ and TYMP on day 0 (Figure 4A), and between IFN-λ and TYMP on day 0, 3, and 7 (Figures 4B,D,F). There was no correlation between IFN-γ and TYMP on day 3 and 7 (Figures 4C,E). These data indicate that TYMP does play a role in virus-mediated reactions and may be a mediator of IFN. Additional studies are needed to understand the detailed mechanism and the cellular sources that are responsible for the SARS-CoV-2 increased TYMP expression.

## Discussion

COVID-19 pandemic is continuingly threatening our life and has globally affected more than 89 million individuals and caused more than 1.9 million global deaths by January 10, 2021 (https://coronavirus.jhu.edu/). Based on WHO's International Clinical Trials Registry Platform (WHO ICTRP), 3,369 COVID-19 associated clinical trials are undergoing or were finished (https://clinicaltrials.gov/ct2/who_table, January 10, 2021 data). Unfortunately, no effective therapeutic strategy has been established (6). By using the MGH Emergency Department COVID-19 Cohort with Olink Proteomics database (22), this study, for the first time, unbiasedly demonstrated that plasma TYMP level is significantly increased and correlated to the acuity, inflammation, D-dimer (evidence of thrombotic events), and organ damage in the COVID-19 patients. Our findings suggest that TYMP is a more sensitive and specific marker for COVID-19 because in the COVID-19 patients with the lowest CRP (<20 mg/L), their TYMP expression was significantly higher than the non-COVID-19 patients, even they have a very high level of plasma CRP (>60–180 mg/L, Figure 2C). This finding is in line with a platelet gene expression study in patients with COVID-19 (27), in which TYMP mRNA was 1.83-fold increase in ICU patients, but it was only 1.45-fold increase in non-ICU patients. The absolute counts of nucleated cells are not associated with TYMP, suggesting that the change of plasma TYMP has not resulted from these cells. However, in a dual-center, two-cohort study, TYMP levels in monocytes of COVID-19 patients were increased in a range of 2.0- to 35-fold, in a monocyte-type dependent manner (Supplementary Table 1) (28). The CD163^hi^ monocytes, which are M2 macrophage-skewed, had the highest TYMP expression (35-fold increase) and the HLA-DR^hi^CD83^hi^ monocytes, which are M1-skewed, had the second high TYMP levels (15.5-fold increase). Interestingly, a recent study indicated that SARS-CoV-2 infects both M1 and M2 macrophages with the same permissivity, but elicits an M2-type genes-enriched response in transcriptional levels (29). These data further indicate that TYMP does participate in the systemic immune response to SARS-CoV-2 infection. However, since the numbers of classical, intermediate, and non-classical monocytes decreased in COVID-19 patients (29), the increase of TYMP in these particular monocyte populations may not account for the increased plasma TYMP. The detailed pathophysiological mechanism behind this should be further studied. Since red blood cells do not express TYMP and TYMP is not a secreted protein (11, 12), we consider that the increased plasma TYMP in COVID-19 patients was from either thrombolysis of the platelet-rich thrombi or organ damage. Both platelet and lung have high TYMP expression.

While there have been no extensive studies clarifying how SARS-CoV-2 affects bone marrow cells, several studies found that SARS-CoV-2 is detectable in the bone marrow (30, 31). Based on the study from Manne et al. (27), we speculate that SARS-CoV-2 can infect megakaryocytes and leads to the production of TYMP-overexpressing platelets. As mentioned above, our recent studies demonstrated that TYMP deficiency or inhibition significantly inhibited thrombosis in mice (12, 13). Currently, no human study has been conducted to clarify the role of TYMP on thrombosis. However, TYMP expression is high in several systemic diseases that are associated with a high risk of thrombosis. It has been reported that increased TYMP expression in human hepatocellular carcinoma correlates with a high incidence of portal vein tumor thrombosis (32). Aspirin, an anti-platelet drug, inhibits TYMP production in a monocyte cell line, THP1 (33), suggesting that aspirin may achieve its anti-thrombotic effect *via* inhibition of TYMP production in platelets. Perfusion of erythrocyte-encapsulated TYMP to mice resulted in thrombus formation in the lungs (34). All these data suggest that TYMP has a mechanistic role in platelet activation and aggregation toward thrombosis.

The rate of thrombosis and hemorrhage in COVID-19 patients is as high as 53 and 16%, respectively (35). Studies have demonstrated that microthrombi present in the lung of COVID-19 patients, and full-dose anticoagulation has been considered as a preventive treatment. Administration of antiplatelet drugs to critical COVID-19 patients has achieved some clinical benefits (35). Our recent study has demonstrated that TPI is a rapid-acting, safe antiplatelet medicine (13). As an auxiliary component of a novel anticancer drug, Lonsurf, TPI was approved by the FDA for clinical use. Lonsurf contains trifluridine and TPI. Trifluridine is a nucleoside analog and has been used as an anti-herpesvirus drug, in addition to its anti-cancer role, in which it acts as a thymidine-based nucleoside metabolic inhibitor. Since TYMP plays an important role in platelet activation, thrombosis, and inflammation, and inhibition of TYMP with TPI significantly inhibited thrombosis in mice without causing bleeding, our study suggests that targeting TYMP with TPI could be a novel regimen for reducing COVID-19-associated thrombotic risk and inflammation. This hypothesis is supported by a recent study, in which TPI was found to bind to the uridine site and inhibited SARS-CoV-2 Nsp15 endoribonuclease function (36).

Taken together, our study, for the first time, indicates that plasma TYMP is correlated with thrombotic events, inflammation, and tissue damages in COVID-19 patients. TYMP may be a novel and sensitive acuity marker for patients with COVID-19. Targeting TYMP with TPI could be a potentially effective therapy for COVID-19.

## Data Availability Statement

The original contributions generated in the study are included in the article/Supplementary Material, further inquiries can be directed to the corresponding author.

## Author Contributions

HY found the MGH COVID-19 Cohort study, extracted TYMP-related data, conducted statistical analyses, literature search, and participated in writing the paper. WL acquired MGH Emergency Department COVID-19 Cohort raw data from Olink, generated figures based on HY's analyses, and wrote the paper. All authors contributed to the article and approved the submitted version.

## Conflict of Interest

The authors declare that the research was conducted in the absence of any commercial or financial relationships that could be construed as a potential conflict of interest.
