# Supplementary material for: Thymidine Phosphorylase Is Increased in COVID-19 Patients in an Acuity-Dependent Manner
Source: Front Med (Lausanne). 2021 Mar 22;8:653773. doi: 10.3389/fmed.2021.653773 (PMC8019714; doi:10.3389/fmed.2021.653773)
Supplement: Supplementary file 2 [file Presentation_1.pdf]

Supplemental Figure I

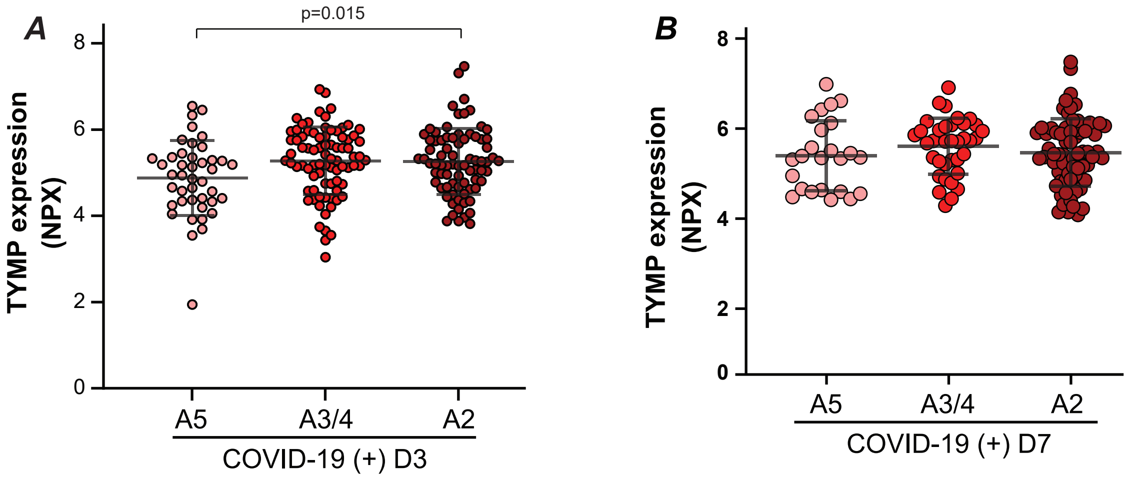

Supplemental Figure II

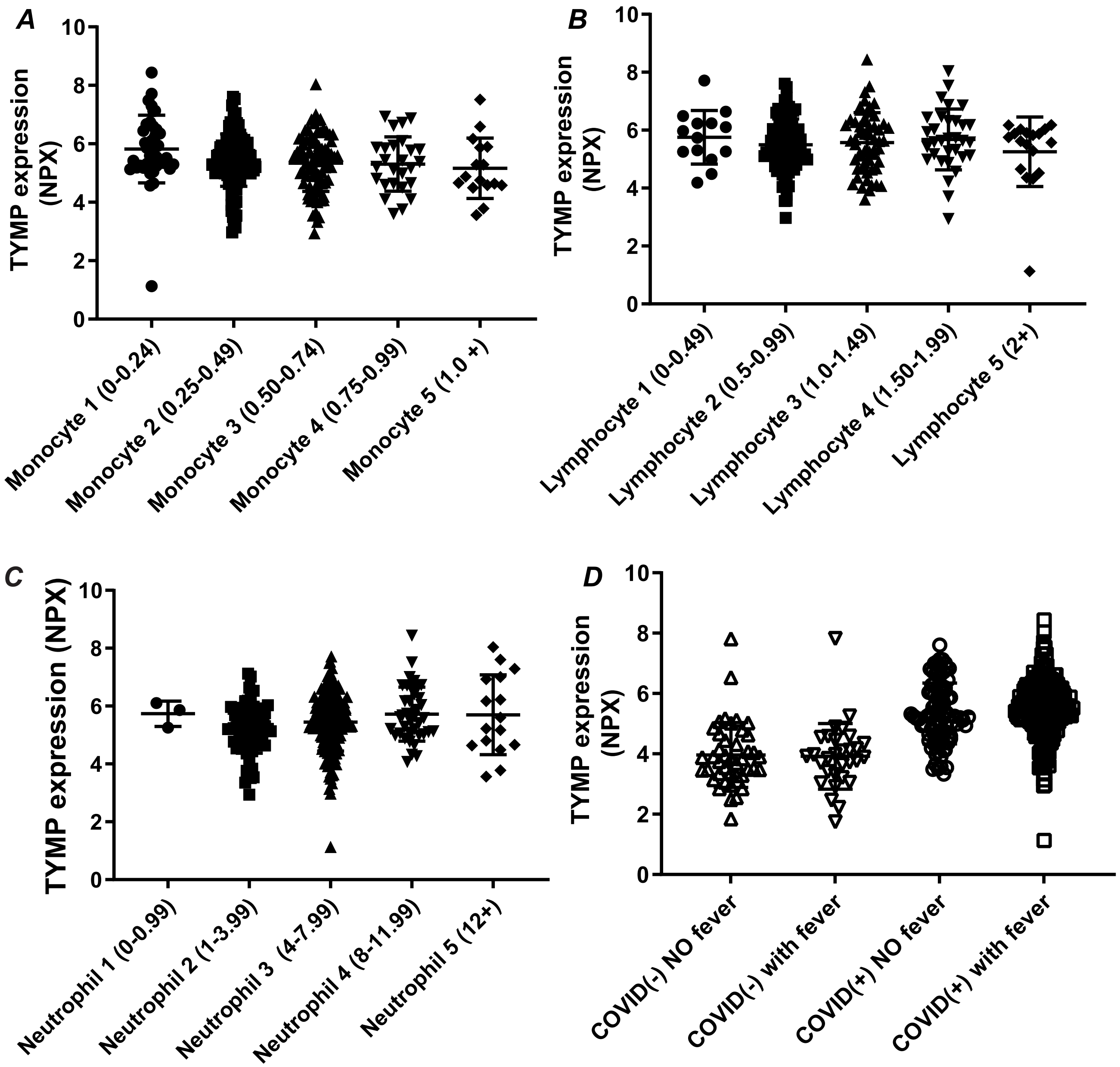

Supplemental Figure III

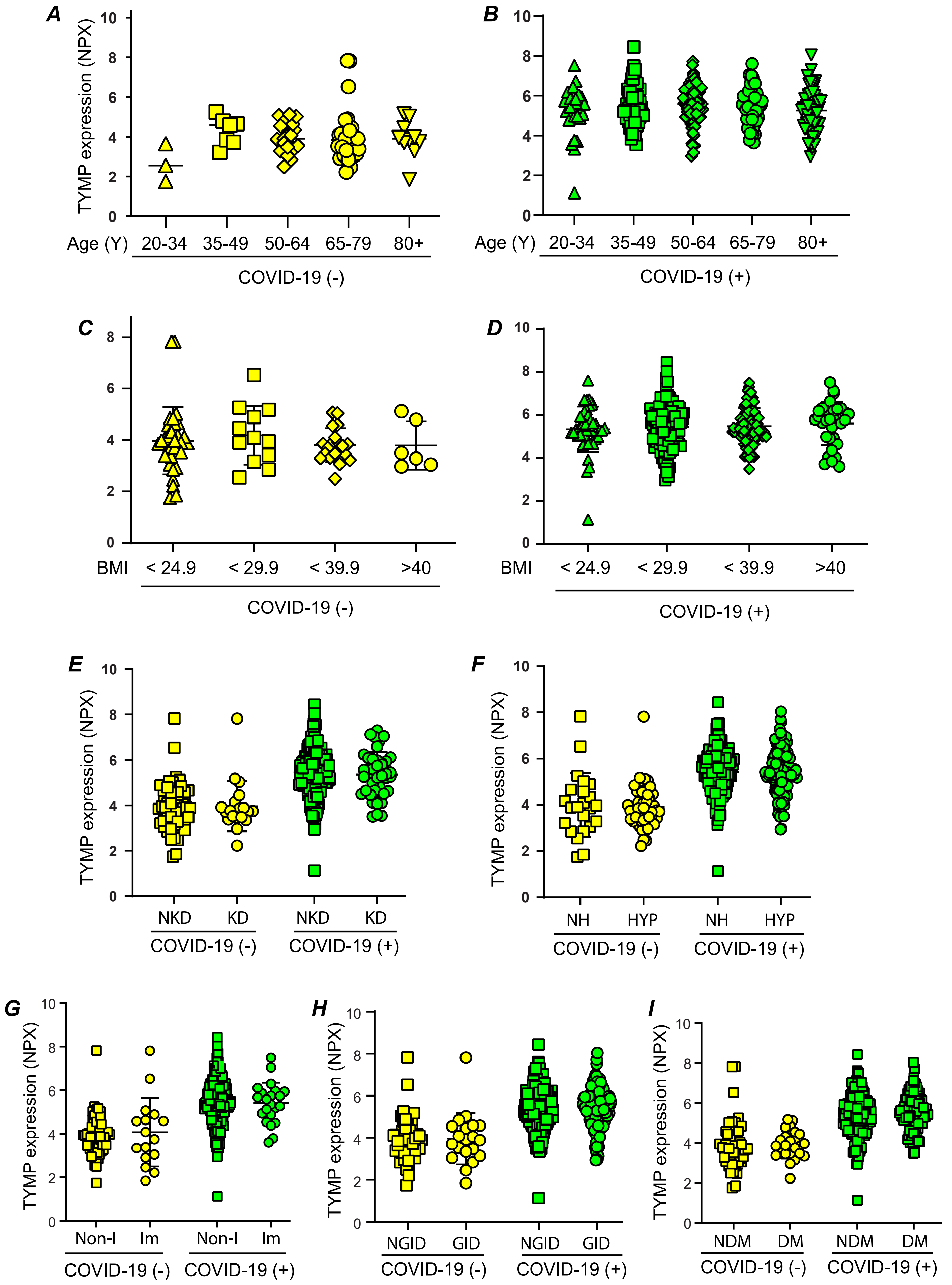

Supplemental Figure IV

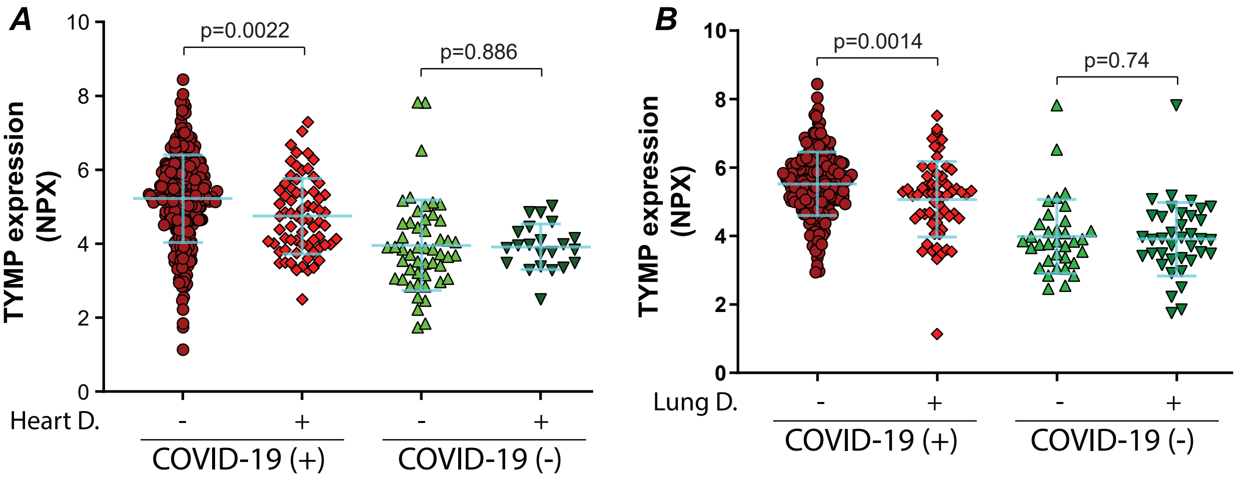

Supplemental Figure V

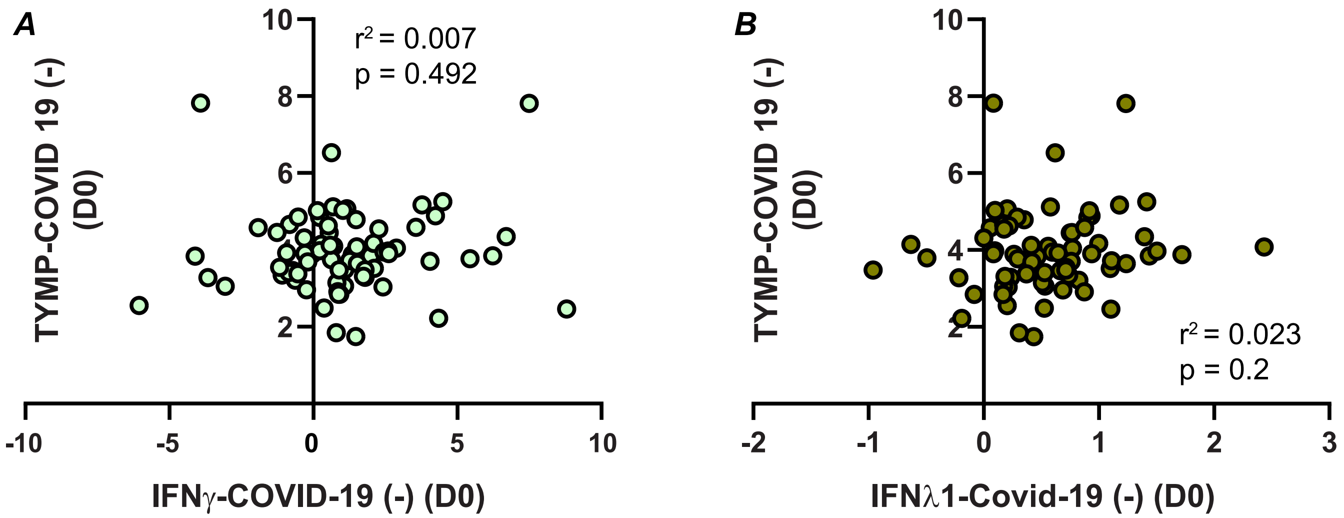

Figure legends for supplemental figures

Supplemental Figure I. TYMP data were sorted based on the acuity on day 3 (**A**) and day 7 (**B**), and then TYMP expression was analyzed based on the defined groups. Acuity (A) 2 = Intubated, ventilated, survived to 28 days, A3 = Non-invasive ventilation or high-flow nasal cannula, A4 = Hospitalized, supplementary O_2 required, and A5 = Hospitalized, no supplementary O_2 required.

Supplemental Figure II. TYMP data were sorted based on cell counts (**A**, **B**, and **C**), fever (**D**), and then TYMP expression was analyzed.

Supplemental Figure III. TYMP data were sorted based on age (**A** and **B**), BMI (**C** and **D**), the presence or absence of kidney diseases (**E**), hypertension (**F**), pre-existing immunocompromised conditions (**G**), pre-existing gastrointestinal diseases (**H**), and diabetes mellites (**I**), and then TYMP expression was analyzed in both COVID-19 negative (yellow) and positive (green) patients. BMI = body mass index, NKD = no kidney diseases, KD = having some type of kidney diseases, NH = no hypertension, HYP = having hypertension, non-I = no pre-existing immunocompromised conditions, Im = having pre-existing immunocompromised conditions, NGID = no pre-existing gastrointestinal diseases, GID = having pre-existing gastrointestinal diseases, NDM = no diabetes mellites, and DM = having diabetes mellites.

Supplemental Figure IV. TYMP data were sorted based on the presence or absence of primary heart (**A**) and lung (**B**) diseases, and then TYMP expression was analyzed.

Supplemental Figure V. Plasma TYMP level is not correlated with the expression of IFN-γ or IFN-λ in non-COVID-19 patients. TYMP, IFN-γ, and IFN-λ data on day 0 were extracted and the correlation between TYMP plasma levels and IFN-γ or IFN-λ was analyzed, respectively.

The table was generated based on supplemental data for Figure 2 of the following paper.			
Severe COVID-19 Is Marked by a Dysregulated Myeloid Cell Compartment, Cell, Volume 182, Issue 6, 17 September 2020, Pages 1419-1440.e23			
https://doi.org/10.1016/j.cell.2020.08.001			
**Cluster**	**Monocyte description**	**LogFC**	**Fold change (FC)**
0	Classic monocytes	0.92	8.3
1	**HLA-DR^{hi}CD83^{hi}**	**1.191**	**15.5**
2	**CD163^{hi}**	**1.543**	**34.9**
3	HLA-DR^{lo}S100A^{hi}	0.492	3.1
4	non-classic	0.758	5.7
21	mixed	0.304	2.0

Table 1. TYMP expression in peripheral-blood monocytes.
